# Discovery of Novel Soluble Epoxide Hydrolase Inhibitors as Potent Vasodilators

**DOI:** 10.1038/s41598-018-32449-4

**Published:** 2018-10-02

**Authors:** Neetika Tripathi, Sarvesh Paliwal, Swapnil Sharma, Kanika Verma, Ritika Gururani, Akanksha Tiwari, Amrita Verma, Monika Chauhan, Aarti Singh, Dipak Kumar, Aditya Pant

**Affiliations:** 1 Department of Pharmacy, Banasthali University, P. O. Banasthali, 304022 Rajasthan, India; 2 Indian Institute of Toxicology Research, Mahatma Gandhi Marg, Post Box No- 80, Lucknow, 226001 UP India

## Abstract

In view of the role of sEH (soluble epoxide hydrolase) in hypertension, we have developed a rigorously validated pharmacophore model containing one HBA (Hydrogen Bond Acceptor), two HY (Hydrophobic) and one RA (Ring Aromatic) features. The model was used as a query to search the NCI (National Cancer Institute) and Maybridge database leading to retrieval of many compounds which were sorted on the basis of predicted activity, fit value and Lipinski’s violation. The selected compounds were docked into the active site of enzyme soluble epoxide hydrolase. Potential interactions were observed between the features of the identified hits and the amino acids present in the docking site. The three selected compounds were subjected to *in vitro* evaluation using enzyme- based assay and the isolated rat aortic model followed by cytotoxicity studies. The results demonstrate that the identified compounds are potent, safe and novel soluble epoxide hydrolase inhibitors.

## Introduction

Despite availability of many drugs for the treatment of hypertension the optimal control of blood pressure is far from reality which may be due to involvement of various factors on the pathogenesis of hypertension and associated diseases.

**Figure 1:**
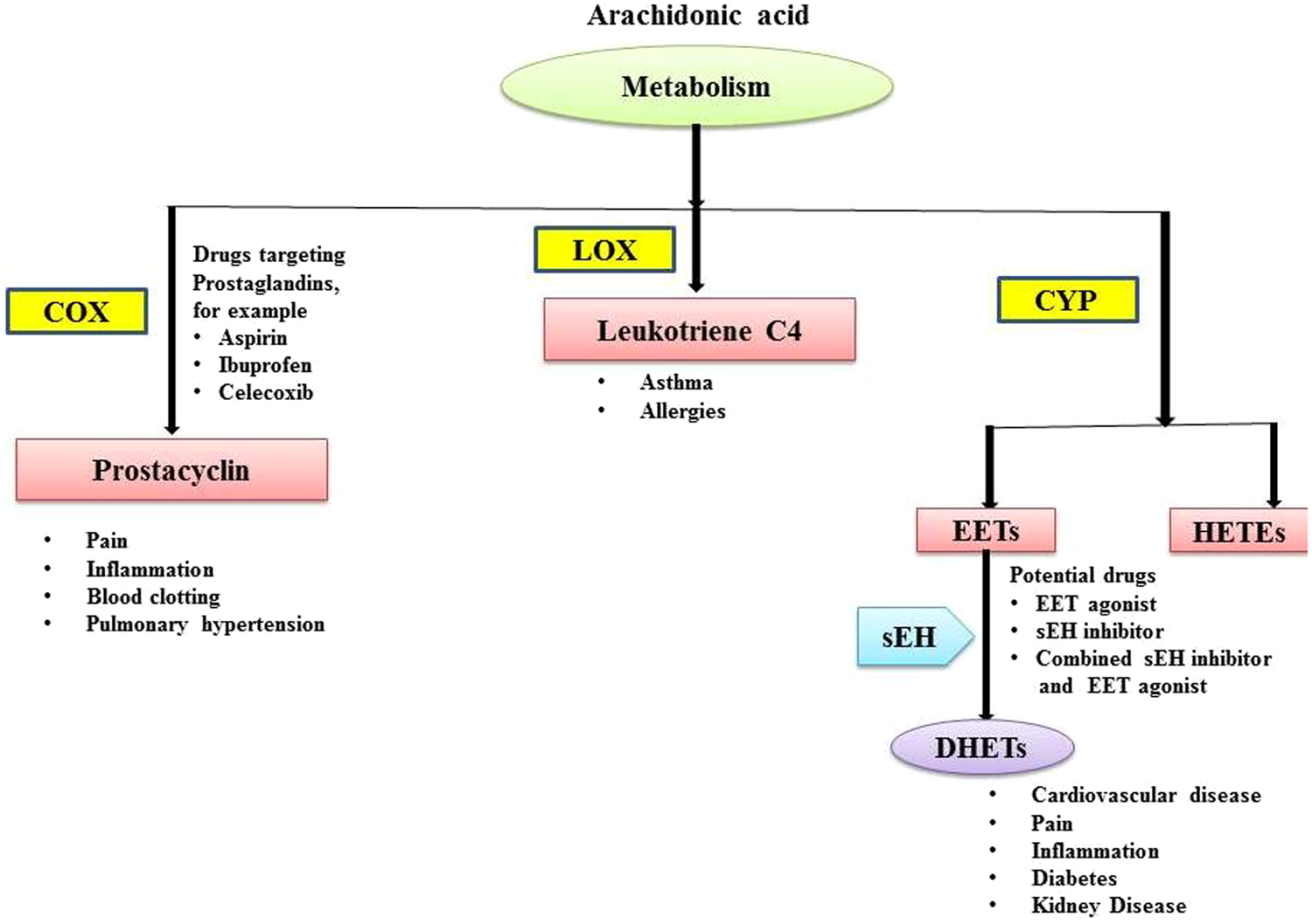
Therapeutic targets in the arachidonate cascade. Three key pathways- the cyclooxygenase (COX), Lipoxygenase (LOX) and cytochrome P450 (CYP) pathways, Epoxyeicosatrienoic acid (EET), Dihydroxyeicosatrienoic acid (DHET).

One of the most promising and emerging targets for the development of antihypertensive drugs is soluble epoxide hydrolase (sEH). Mammalian tissues like liver, kidney, intestine and vessels show highest activity of this enzyme. The sEH belongs to α/β-hydrolase family of enzyme exhibiting high level of selectivity for epoxides of fatty acids. Epoxyeicosatrienoic acids (EETs) that are epoxides of arachidonic acid are responsible for vasodilation in various renal, mesenteric, cerebral, pulmonary & coronary vascular tissues^1^. These EETs are converted into dihydroxyeicosatrienoic acids (DHETs) in the presence of sEH enzyme and it is important to note that DHETs are devoid of vasodilatory action^2^. In view of potential role of sEH in diminishing the EET induced vasodilation, efforts have been made to inhibit this enzyme^3^ (Fig. 1).

Epoxides containing compound were the first developed inhibitors of sEH enzyme but they only showed *in-vitro* activity and found to be ineffective in cell culture and *in vivo* studies^4,5^. Further urea, carbamate & amide derivatives appeared to be good inhibitor of the enzyme and noticeably these compounds showed satisfactory *in vivo* activity^6^. With the help of ligand and structure based drug design technique the chemical structure of these compounds were further modified to produce more potent compounds^7–10^. Esters and salts of adamantane-1-yl-ureido]-dodecanoic acid (AUDA) have been found to be good inhibitor of sEH but its clinical use has been restricted due to metabolic instability & limited solubility in water and many organic solvents^7,10,11^. To date, very few soluble hydrolase inhibitors have been developed and evaluated pre-clinically and some are in pipe line of clinical trial. For instance, two of the inhibitors, namely AR9281 and GSK 2256 294 have already showed promising effects in phase 1 human clinical trials with minimum toxicities. In addition, GSK 2256294 has demonstrated to improve endothelial dysfunction in obese males with chronic obstructive pulmonary disease (COPD). Considering the definite role of soluble epoxide hydrolase in management of hypertension, in the present study exhaustive efforts have been made to develop more promising molecules as soluble hydrolase inhibitor to address hypertension in better means.

Notably, till date there is no commercial drug available as soluble hydrolase inhibitor and hence there is an urgent need to develop novel inhibitors that could able to reduced cardiovascular diseases and associated mortalities at an impressive rate. The drug design techniques such as ligand based and structure-based optimization of the chemical structures led to more potent compounds. In view of this, we performed 3D QSAR based pharmacophore modeling, database mining and molecular docking in conjugation with biological evaluation to discover novel soluble epoxide hydrolase inhibitors with potential for their future development as potent antihypertensive agents.

## Results

### Pharmacophore generation

**Figure 2:**
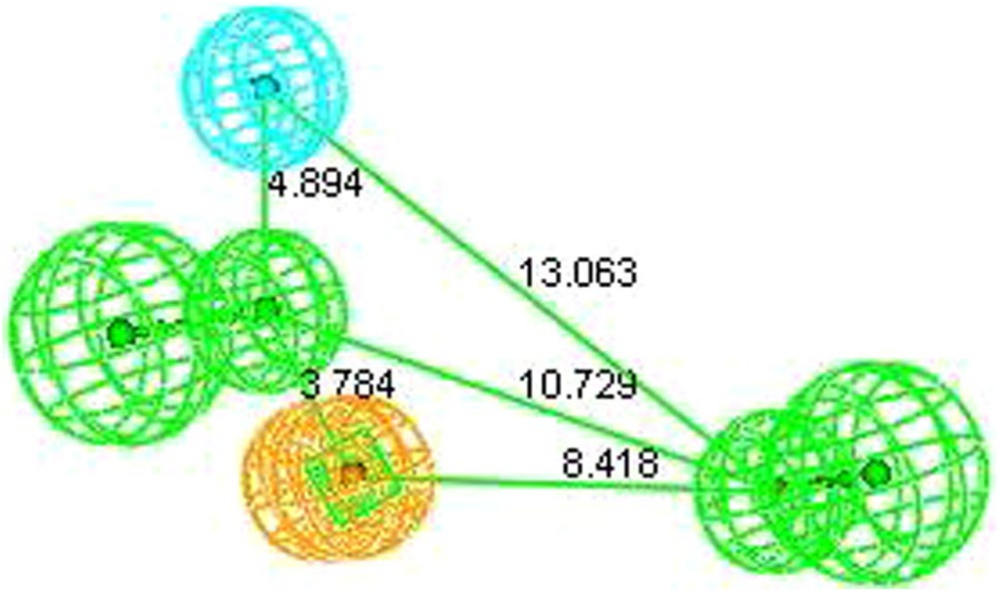
Pharmacophore with two HBA, one HY and RA features.

Conformational analysis of all the selected training set compounds was carried out by choosing the best flexible conformation option available with Discovery Studio (v2.0), keeping an energy threshold of 20.0 kcal/mol above the global minimum energy in both torsional and cartesia. The best flexible search has been opted because in contrast to fast method it has the ability to explore the low energy areas of the conformational space and can generate conformations that donot relates to a local energy minima. Moreover, best method can easily reproduce the ligand bound conformation of the chosen compound. Before the development of 3D QSAR based pharmacophore (hypogen) models, common-feature pharmacophore (Hip Hop) models were constructed to recognize the important features, and this led to identification of 2 HBA, 1 HY and 1 RA feature (Fig. 2).

**Figure 3:**
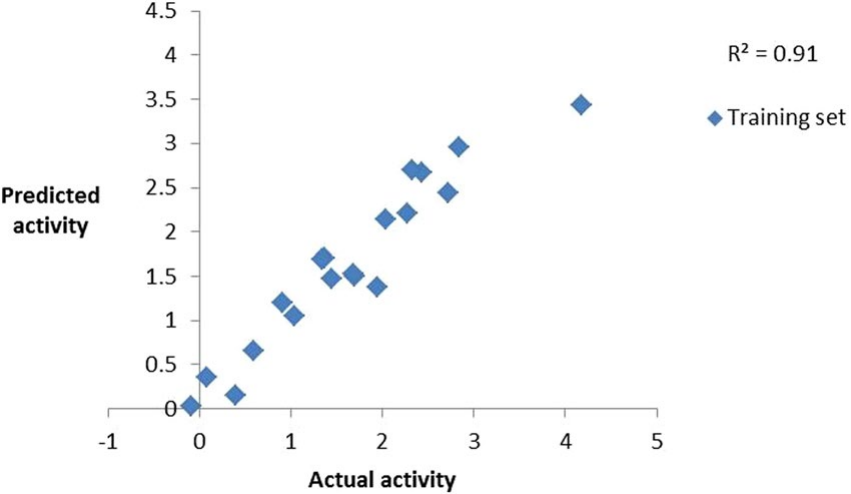
Observed correlation coefficient values of training set.

Taking into account the aforementioned features different 3D QSAR based pharmacophore (Hypogen) models were constructed. During the modeling it was observed that compounds 9 showed ahigh error ratio, eventually it was removed from the dataset with an aim to further enhance the quality of the model. This kind of behavior of compound 9 indicates typographical error or inappropriate experiment observation or may be different mechanism of action^12^. Many pharmacophore models were generated and statistically evaluated. Finally, hypothesis 1 comprising of 2 HBA, 1 HY and 1 RA with root mean square (RMS) of 0.62, correlation coefficient of 0.95, weight of 0.94 and configuration of 12.64 was considered as best (Table S1) (Fig. 3).

**Figure 4:**
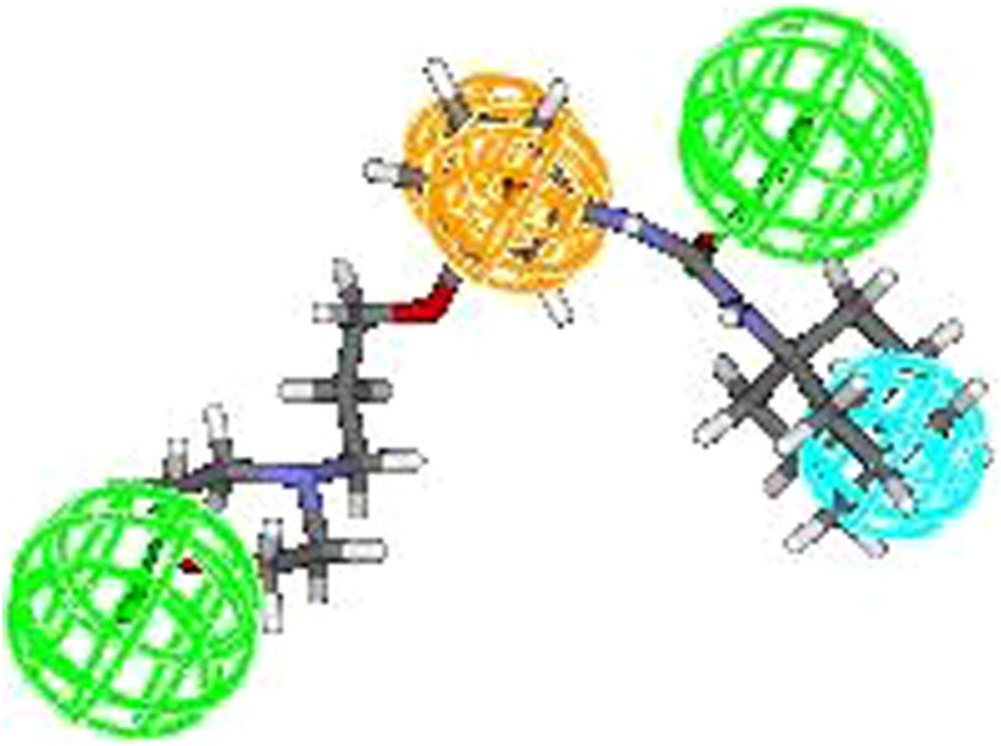
Most active compound of training set.

**Figure 5:**
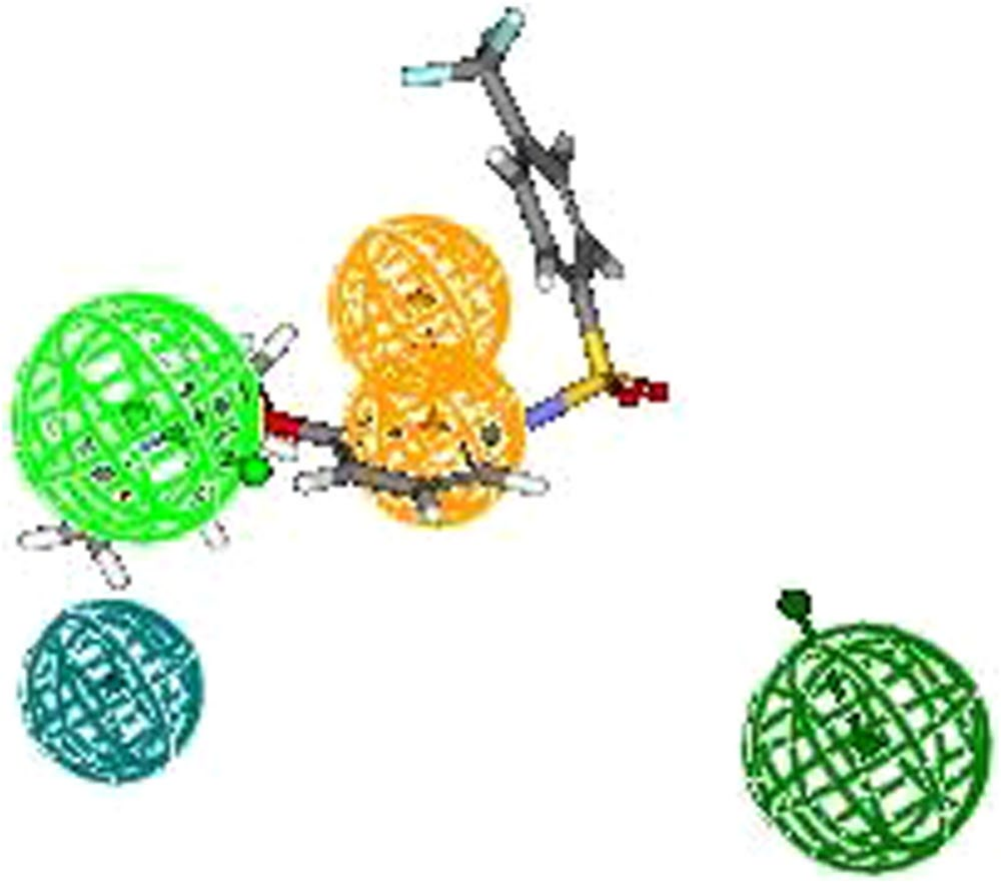
Least active compound of training set.

All the training set compounds employed in model construction were subjected to pharmacophore mapping and it was observed that most active compound (5a) of the training set fully mapped all the chemical features (Fig. 4) whereas the least active compound (7) clearly missed one HY and one HBA feature (Fig. 5). The results of pharmacophore mapping clearly shows that the model has the capability of differentiatingbetween active and inactive sEH inhibitors on the basis of identified features and their distance.

In view of good correlation coefficient value and mapping pattern, the model was subjected to rigorous validation using a battery of tests.

### Fischer’s randomization test

**Figure 6:**
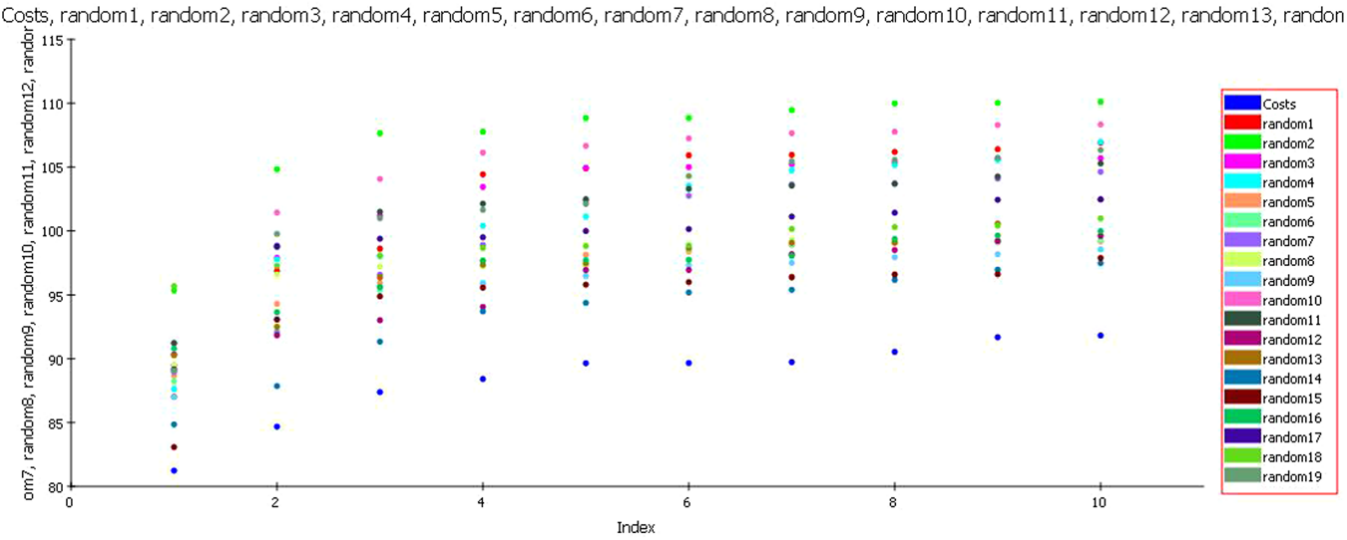
Fischer’s randomization test.

A confidence level of 95% was selected during Fischer’s randomization test and during the process in total nineteen random spreadsheets were produced employing the chemical features and parameters that were used in the construction of the model (Fig. 6). The total cost of all the models generated during the process of Fischer’s randomization was observed to be high when compared to the total cost of the selected pharmacophore model. This clearly shows that the model is not a result of chance correlation; rather it is depicting true correlation statistics.

### Rm^2^ matrices

With an aim to determine the closeness between estimated and actual activities, the rm^2^ metrics (average rm^2^ and delta rm^2^) developed by Roy *et al*. was computed^13^. The average rm^2^ for the training set was observed to be 0.83 whereas the delta rm^2^ was found to be 0.07. The prescribed values of ‘Average rm^2^’ and ‘Delta rm^2^’ are >0.5 and <0.2.

### Internal and external test set prediction

**Figure 7:**
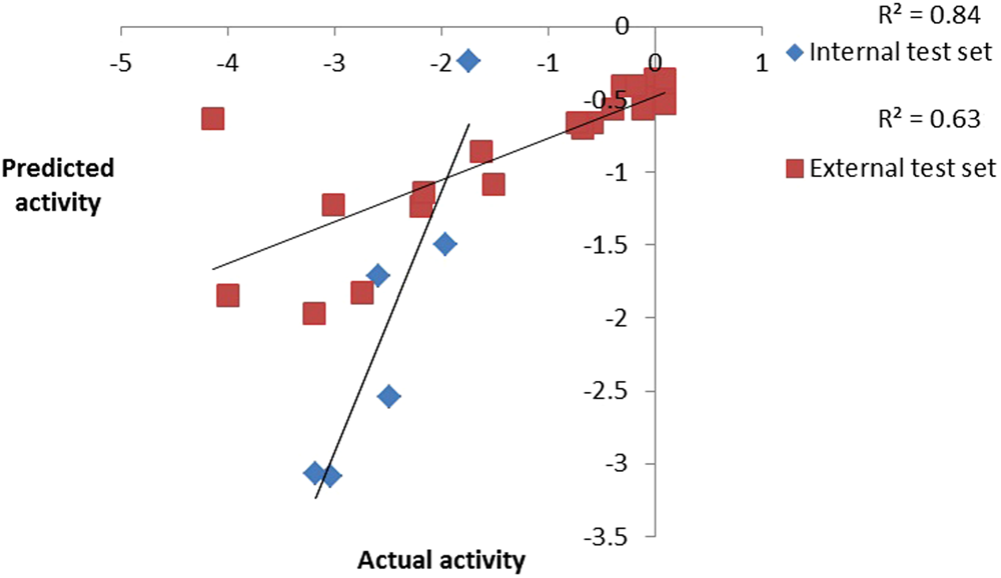
Observed correlation coefficient values of internal and external test set.

**Figure 8:**
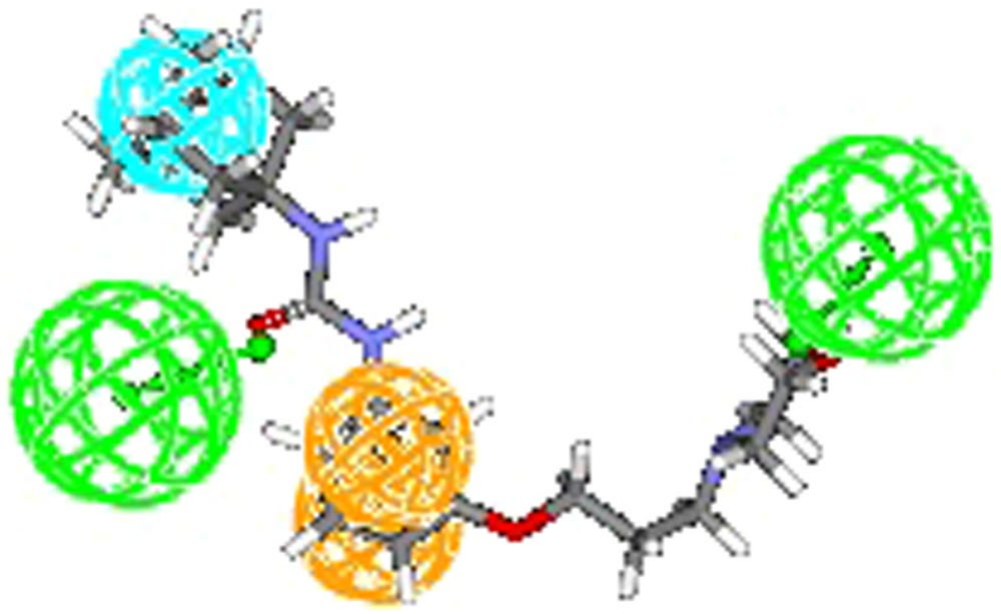
Most active compound of the external test set exhibit a perfect four feature mapping.

A pharmacophore model is only considered useful when it has the ability to precisely estimate the biological activity of compounds not part of the training set. Hence, the model was used to estimate the activities of 6 internal and 21 external test set compounds belonging to class of unsymmetrical non-adamantyl N,N′-diaryl urea and amides derivatives^14^. The observed correlation coefficient values 0.84 and 0.63 for the chosen test sets as shown in (Fig. 7) which clearly indicates the high predictability, correctness and universality of the model. As presumed most active compound of the external test set showed a perfect four feature mapping (Fig. 8).

### Güner-Henry Scoring Method

The Guner-Henry scoring method was used to evaluate the virtual screening ability of the pharmacophore model. Parameters like %yield of actives, enrichment factor, false positives and goodness of hit score (GH scoring method) were calculated and evaluated. In total 238 actives were retrieved from the data set of 311 compounds. The model exhibited 90.51% yield with enrichment factor of 1.18. The false positives appeared to be just 11%. On the whole the model showed a GH score of 0.67 which is well within the recommended limit of GH score (≥0.60). The findings of Guner-Henry scoring clearly showed the capability of the model to correctly estimate the activity of the most of the compounds (Table S2).

### Virtual screening

**Figure 9:**
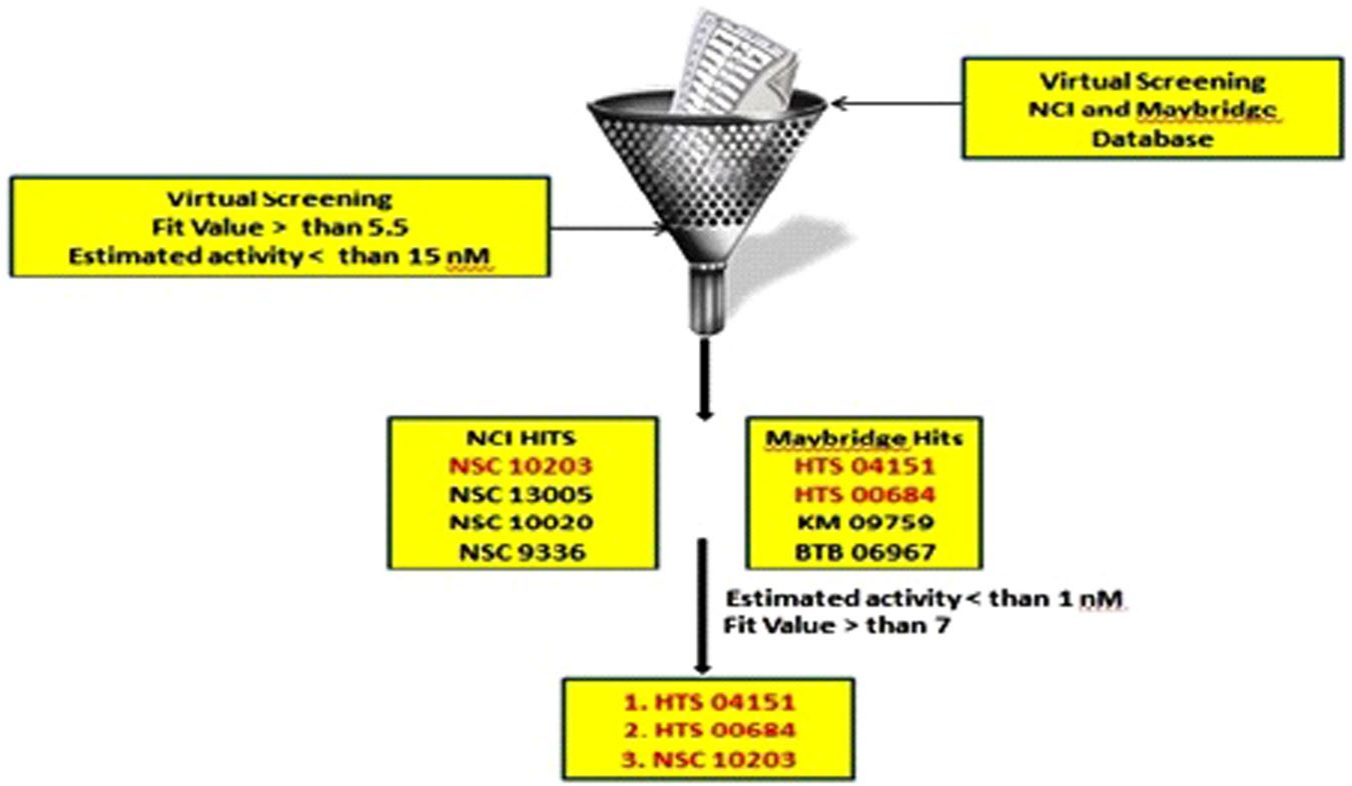
Ligand driven pharmacophore based virtual screening.

In view of good GH score, the developedmodel was used as a query to search the NCI and Maybridge database containing 2, 38, 819 and 53,000 structures. Total 297 hits from NCI and 211 hits from Maybridge were retrieved which were sorted on the basis of Lipinski’s rule of five. The remaining hits were screened on the basis fit value (greater than 5.5) and estimated activity (less than 15 nM), this lead to retention of eight hits from NCI and Maybridge database (NSC 10203, NSC 13005, NSC 10020, NSC 9336, HTS 04151, HTS 00684, KM 09759 and BTB 06967). With an aim to further reduce the list, the identified hits (Table S3) were further screened on the basis of predicted activity <1 nM and fit value >7. Finally three hits (HTS 04151, HTS 00684 & NSC 10203) with very high potency and fit value (Fig. 9) were chosen for sEH enzyme based assay and rat aortic ring based vasodilation assay but prior to the all the three compounds were docked into the active site of sEH to understand the molecular interaction between the functional groups of the hits and active site amino acids.

### Molecular docking

**Figure 10:**
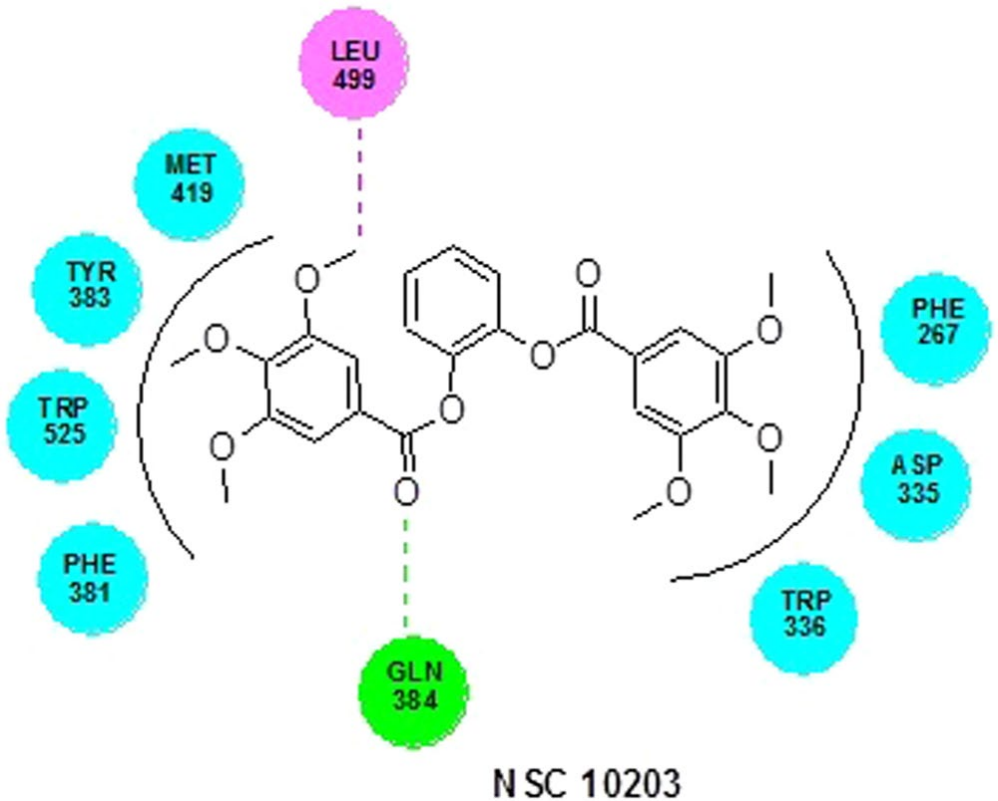
Docked conformation of NSC 10203 on the active site of soluble epoxide hydrolase.

**Figure 11:**
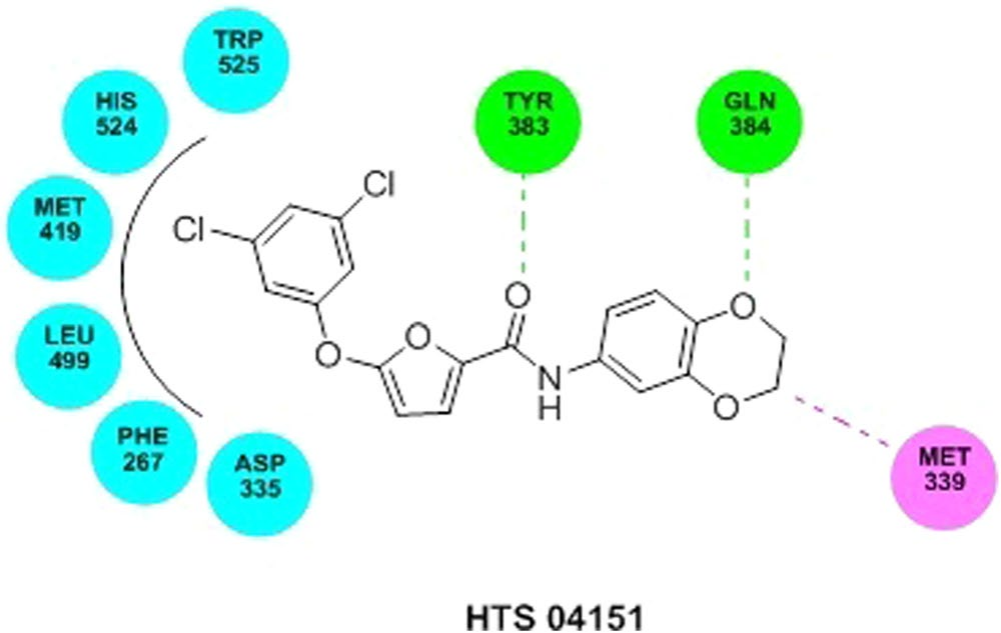
Docked conformation of HTS 04151on the active site of soluble epoxide hydrolase.

**Figure 12:**
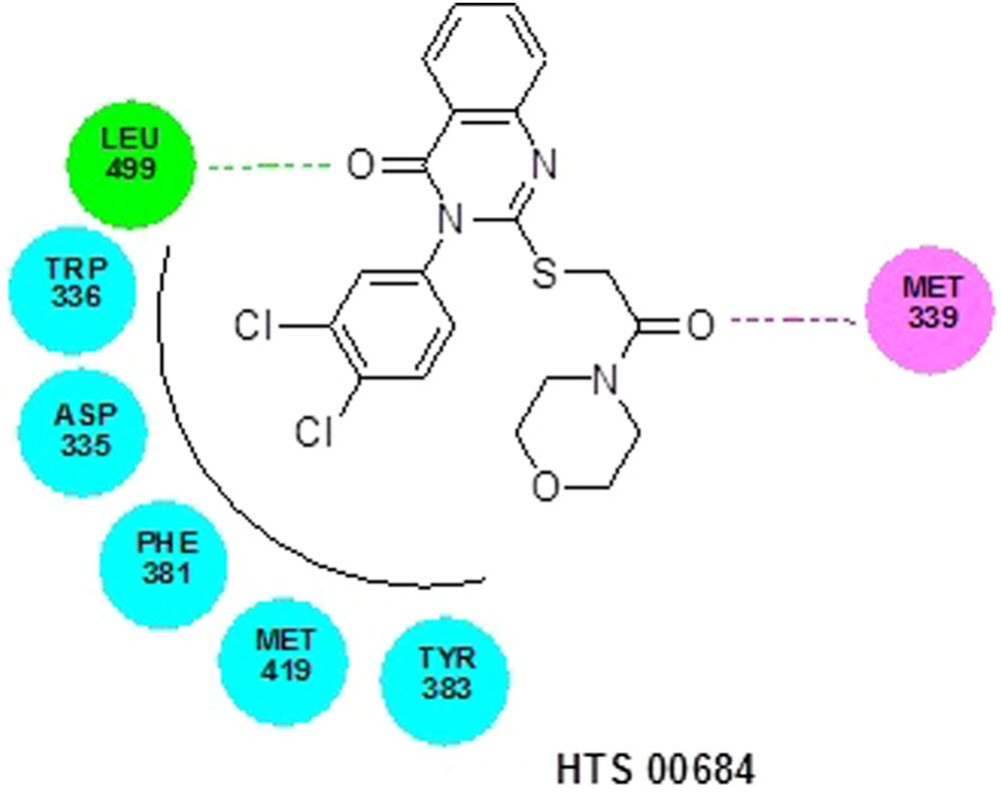
Docked conformation of HTS 00684 on the active site of soluble epoxide hydrolase.

The LibDock score of NSC 10203 was observed to be 133.26 and it exhibited one hydrogen bond interaction, one van der waal interaction and seven hydrophobic interactions. The oxygen of the carbonyl group present in the structuring of NSC 10203 showed hydrogen bonding with GLN 384 whereas LEU 499, showed Van der Waal interaction with methyl group. The hydrophobic skeleton of NSC 10203 was surrounded by MET 419, TYR 383, TRP 525, PHE 331, TRP 336, ASP 335, PHE 267 (Fig. 10). HTS 04151 also docked very well with LibDock 116.08 and exhibited two hydrogen bond interaction, one van der waal interaction and six hydrophobic interactions. Hydrogen bond interaction was observed among amide oxygen and TYR 383 and oxygen of dioxane ring and GLN 384. MET 339 showed Van der Waal interaction with of dioxine. HTS 04151 showed hydrophobic interaction with MET 419, HIS 524, TRP 525, LEU 499, PHE 267 and ASP 335 (Fig. 11). HTS 00684 with LibDock score of 80.91 showed one hydrogen bond, one Van der Waal and five hydrophobic interactions. Oxygen of the carbonyl group showed hydrogen bond interaction with LEU 499. MET 419 showed Vander Waal interaction with oxygen of the carbonyl group. HTS 00684 showed hydrophobic interaction with ASP 335, TRP 336, PHE 381, MET 419 and TYR 383 (Fig. 12).

### Biological Evaluation

#### sEH inhibitory assay

**Figure 13:**
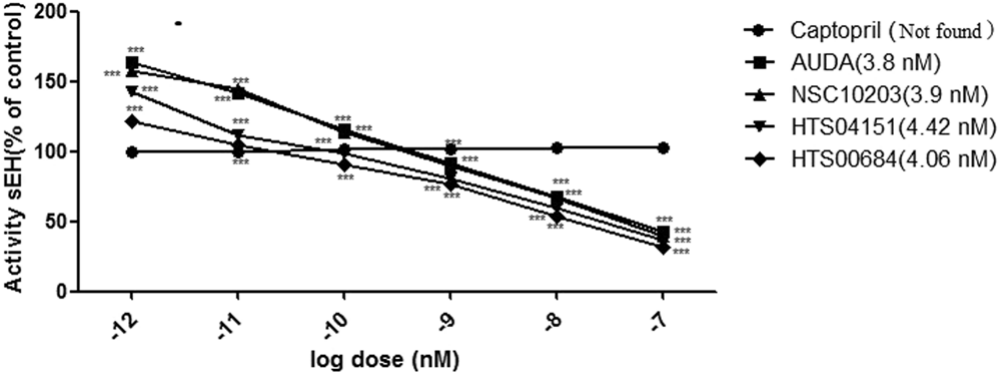
Concentration dependent inhibition of human sEH by compounds (**a**) AUDA (**b**) NSC 10203 (**c**) HTS 04151 (**d**) HTS 00684; n = 3.

Results of enzyme-based assay of test compounds NSC 10203, HTS 04151 and HTS 00684 reveals that all the three hits possess potent sEH inhibitory activity (Fig. 13). Captopril, was used as negative control which is devoid of any sEH inhibitory activity. It is noticeable that compound NSC 10203 exhibited equal potency to standard AUDA.

#### Rat aortic ring-based vasodilation assay

**Figure 14:**
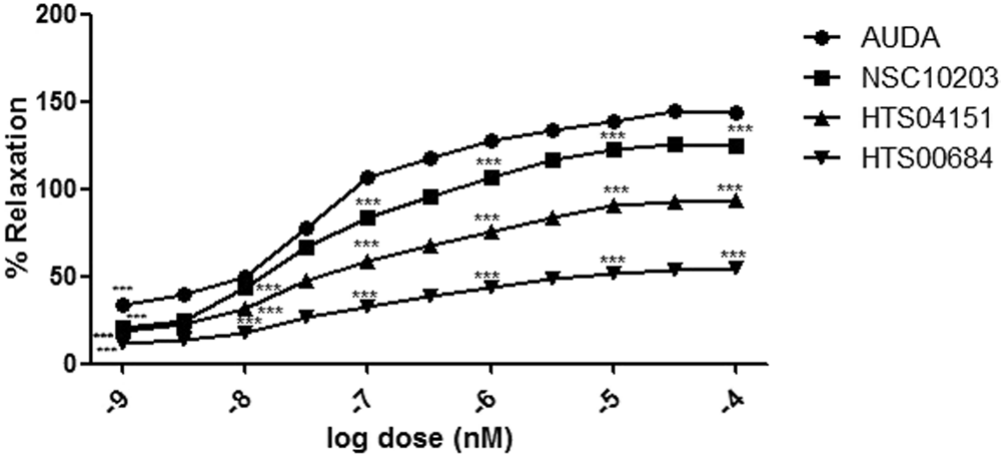
Vasodilatory effect of (**a**) AUDA (**b**) NSC 10203 (**c**) HTS 04151 (**d**) HTS 00684 at various concentrations.

In view of promising sEH enzyme inhibitory activity, all the three lead compounds (NSC 10203, HTS 04151 and HTS 00684) were evaluated for vasodilation activity on rat aortic ring using earlier reported *in-vitro* vasodilation assay^15^. The vasodilation was recorded as the remaining % of relaxation after the addition of all three compounds to pre-contracted rings. Results of study *in-vitro* vasodilation assay revealed that NSC10203, HTS04151 and HTS 00684 have caused dose dependent relaxation, similar to standard AUDA (Fig. 14). NSC 10203 appeared to be most potent compared to AUDA followed by HTS 04151 and HTS 00684. Effective concentration (EC_50_) of AUDA, NSC10203, HTS04151 and HTS 00684 was found as 3.88 nM, 4.34 nM, 4.56 nM and 4.89 nM respectively. These results clearly indicated that all the identified compounds are having good vasodilator activity.

#### *In vitro* cytotoxicity assay

**Figure 15:**
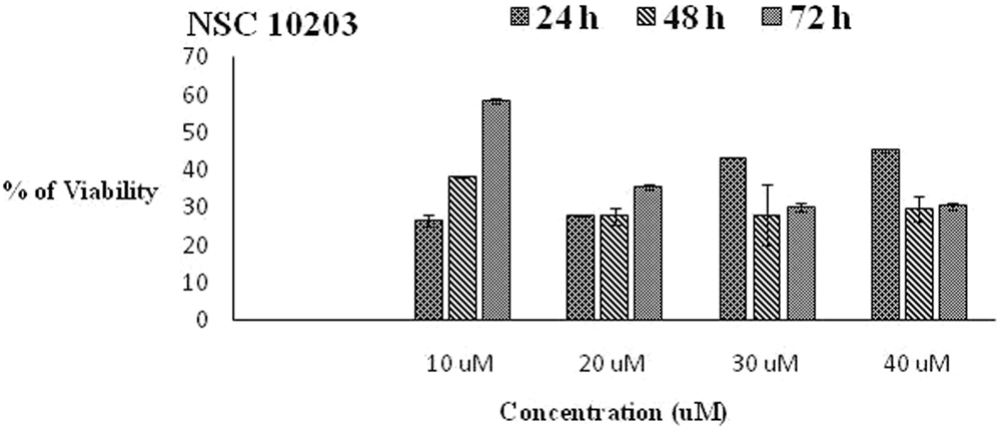
*In- vitro* cytotoxicity assay (**a**) 24 hrs (**b**) 48 hrs (**c**) 96 hrs.

Since, NSC 10203 showed highest potency during sEH enzymatic and *in vitro* vasodilation activity, it was subjected to *in-vitro* cytotoxicity assay to assess its safety for further analysis. Standard end point MTT assay has been used for the study, where hWJ-MSCs served as appropriate test system^16^. hWJ-MSCs are proliferating human stem cells which can get differentiated into adipocyte, chondrocyte and osteocyte lineages. MTT reduction is usual in proliferating cells such as mesenchymal stem cells. Results of cytotoxicity assay are depicted in (Fig. 15). NSC 10203 showed maximum cell survivability, after 48 h and even the cell survivability was not decreased with increase in exposure time (72 h).

## Discussion

Epoxyeicosatrienoic acids (EETs) are the important components of arachidonic acids pathways and they produce prominent vasodilatation through calcium activated potassium channels. Metabolic conversion of active EETs to physiologically inactive dihydroxyeicosatrienoic acids (DHETs) is carried out by the enzyme sEH and this enzymatic conversion deprives the human body for the beneficial cardiovascular effects of the EETs^1^. Thus inhibition of sEH has been considered as a crucial step in producing vasodilation.

In view of this, we initiated the search for novel, structurally diverse and safe sEH inhibitors through sequential application of *in-silico* and *in-vitro* protocols. To start with a data set of sEH inhibitors was chosen to develop quantitative pharmacophore model (Table S4). Utmost care was exercised to include diverse compounds in both training (Table S5) and test set (Table S6) for development of statistically sound model. Before construction of (quantitative) hypogen models, a qualitative Pharmacophore (Hip Hop) model was generated. Hip Hop models are always useful because they provide an early insight into the chemical features responsible for binding of the selected set of compounds. The features appeared in the Hip Hop models were two hydrogen bond acceptors, one hydrophobic feature and one ring aromatic feature (Fig. 2). During the hypogen model development using training set compoundsit was observed that the features appearing in quantitative Pharmacophore model are similar to that of Hip Hop features. In view of commonality of the features between both the models, the selected quantitative pharmacophore model was validated to check its statistical fitness. The results of Fischer’s randomization test, internal test set prediction, external test set prediction, Rm^2^ matrics^13^ and GH scoring^17^ clearly highlights the soundness and universality of the developed quantitative model.

In the second phase of study the validated quantitative model was used to search the NCI and Maybridge databases which stores large number of diverse scaffolds. In order to select a handful of sEH inhibitors, the hits obtained (1508) from virtual screening were carefully sorted on the basis of fit value, estimated activity and Lipinski’s violation in a phase wise manner. The structures of the selected hits are given in Table S3. A close inspection of the structures and their estimated activities led to the selection of three hits (NSC 10203, HTS 04151 and HTS 00684) which are sufficiently diverse and theoretically potent (estimated activity and fit value). In view of their promising activity and structural diversity they were subjected to *in vitro* studies i.e she inhibitory assay, rat aortic ring based vasodilatory assay and safety evaluation but prior to that all the three hits were docked into the active site of the sEH enzyme. The results of the docking show the importance of tryptophan, tyrosine, phenyl alanine, aspartic acid and methionine in binding of NSC 10203, HTS 04151 and HTS 00684 to the active site of sEH. The results are in agreement to the earlier studies on docking of sEH inhibitors^18^. Thepreviously published reports clearly highlights the importance of phenyl alanine and tryptophan in ring aromatic interaction. Earlier reports also reveal the involvement of tyrosine in hydrogen bonding and role of methionine in hydrophobic interactions. The docking poses of NSC 10203, HTS 04151 and HTS 00684 also compliments the pharmacophoric features appeared in the Hip Hop and Hypogen models i.e ring aromatic, hydrophobic and hydrogen bonding.

The results of sEH enzyme inhibitory assay performed using microplate reader also appeared to be promising. Out of all the three hits NSC 10203 showed highest inhibitory potential. It is also noticeable that NSC 10203 exhibited equal potency to that of standard drug AUDA at all the six concentrations (0.001 nM, 0.01 nM, 0.1 nM, 1 nM, 10 nM, 100 nM) (Fig. 13). The results of sEH inhibitory activity are also in agreement to the estimated activity (0.98 nM) and LibDock Score (133.26) of NSC 10203, which is highest among all the compounds. Since all the three compounds exhibited good activities in sEH inhibitory assay they were subjected to *in vitro* vasodilatory assay using rat aortic ring. Here also NSC 10203 was found to be most potent followed by HTS 04151 and HTS 00684 (Fig. 14).

Safety has always been the prime concern during the entire cycle of drug design and development. In view of promising activity of all the three hits and in particular of NSC 10203, an *in vitro* cytotoxicity assay was performed to assess its safety. From the results shown in Fig. 15 it is clear that NSC 10203 offers maximum cell survivability proving its safety even after 72 hrs of exposure time.

Among all the three hits NSC 10203 showed very good *in vitro* activity in both the assays and also appeared to be safe. It is also important to note that NSC 10203 is also a novel compound as revealed by the Tanimoto Similarity Index of 0.13.

Since sEH inhibitors also have anti-inflammatory potential as revealed by previous studies they could be of immense benefit to the patients of CVD because in hypertension and other cardiac ailments there is a definite role of inflammation in the progression of disease^19^.

## Conclusions

Through our well defined *in-silico* workflow viz ligand-based pharmacophore modeling, virtual screening and molecular docking, three potent sEH inhibitors were discovered. The *in-vitro* studies confirmed the sEH inhibitory potential and vasodilation activity of the identified compounds. The cytotoxicity studies revealed the safety of the compounds. The results of the present study clearly show that the discovered compounds have the potential for their development as anti-hypertensive agents.

## Experimental Section

### Generation of pharmacophore model

A data set of 26 sEH inhibitors with wide range of activity (0.8 nM to 15000 nM) and structural diversity was selected and split into training and test set containing 20 and 6 compounds each. Utmost care was taken to ensure structural diversity and wide range of activity value (highly active, moderately active, inactive) while dividing the compounds. The chemical structures of all the compounds under consideration were sketched using ChemDraw Ultra 8.0 and imported in 3D window and subjected to energy minimization using CHARMm force field. A maximum of 255 diverse conformers were generated with the energy threshold of 20 kcal mol^−1^ using diverse conformation generation protocol (best conformer generation) option as provided in Discovery Studio version 2.0^20^.

With an aim to identify the important features responsible for the activity of the training set compounds, common feature pharmacophore models were developed. This process led to identification of hydrogen bond acceptor (HBA), hydrophobic (HY) and ring aromatic (RA) features which were used for the construction of quantitative (hypogen) pharmacophore models. Out of various generated models the best one was chosen on the basis of cost-function analysis, root mean square deviation (RMSD) and correlation coefficient value.

### Validation of generated model

The selectedpharmacophore model was validated using internal and external test set prediction, Fischer’s randomization, Rm2 metrics and GH score.

#### Internal and external test set prediction

The internal test comprised of compounds with high, moderate and poor activity. In addition to internal test set, an external test set comprising of 21 compounds with structural and biological activity diversity was also selected from the previously published literature^14^. The actual vs predicted activity of the internal and external test set compounds were compared to assess the prediction capability and universality of the pharmacophore model.

#### Fischer’s randomization test

Fischer’s randomization test was performed at 95% confidence level. In total 19 random spreadsheets were generated using the features and parameters which were employed to construct the best pharmacophore model. The results obtained from Fischer’s randomization were evaluated to exclude the possibility of chance correlation.

#### Rm2 metrics test

With an aim to observe the difference between predicted and actual activities of the soluble epoxide hydrolase inhibitors Rm^2^ Metrics was calculated. The prescribed values of ‘Average rm2’ is >0.5 and ‘Delta rm2’ is <0.2^13^.

#### Güner-Henry Scoring Method

The applicability of any pharmacophore model lies in its ability to discriminate between the active and inactive compounds. The Güner-Henry (GH) scoring method is frequently used to determine the virtual screening ability of the pharmacophore models. ^18^The Güner-Henry scoring method is often considered as an appropriate metric since it includes the calculation of the percent yield of actives in a database (%Y, recall), the percent ratio of actives in the hit list (%A, precision), the enrichment factor (E) and GH score. These parameters are computed using Eqs 1–4.

1 $$ \% {\rm{A}}={\rm{Ha}}/{\rm{A}}\times 100$$

2 $$ \% {\rm{Y}}={\rm{Ha}}/{\rm{Ht}}\times 100$$

3 $${\rm{E}}={\rm{Ha}}/{\rm{Ht}}$$

4 $${\rm{GH}}=({\rm{Ha}}(3{\rm{A}}+{\rm{Ht}})/4{\rm{HtA}})\times (1-{\rm{Ht}}-{\rm{Ha}}/{\rm{D}}-{\rm{A}})$$

%A is the percentage of known active compounds retrieved from the database (precision); Ha, is the number of actives in the hit list (true positives); A, is the number of active compounds in thedatabase; %Y, is the percentage of known actives in the hit list (recall); Ht, is the number of hits retrieved; D, is the number of compounds in the database; E, is the enrichment of active compounds in the virtual screening hit list in comparison to the non-filtered database and GH is the Güner-Henry score. Since GH scoring is considered as a potential tool in ascertaining the validity of the pharmacophore model, a data set of 311 structurally diverse known soluble epoxide hydrolase inhibitors from six publications^14,21–25^ was made and used to compute different factors associated with GH scoring method.

### Database search

Since the chosen pharmacophore model displayed all the characteristics of a statistically sound model it was used to search the NCI and Maybridge database. The hits retrieved were screened on the basis of estimated activity and fit value. In addition to this, Lipinski’s rule of five was also applied to ensure good oral bioavailability of the selected hits.

### Molecular docking

LibDock module of DS v2.0 (Catalyst, Accelrys Software) was used to analyze the bindingmode of retrieved hits into the active site of sEH enzyme^15^. The reason for choosing LibDock has been its ability to dock into the hot spots (polar and non polar interaction sites) of the receptor and less computation time. During the process ligand was kept flexible while the target protein was kept rigid. The crystal structure of enzyme sEH was obtained from protein data bank (PDB ID: 4JNC). The water molecules were removed and the hydrogen atoms were added. The radius of 10 Å from the geometric centroid of the ligand was defined in the active site. The hits obtained from database search were docked in the active site and the top ten poses were considered for analysis of type of interaction.

### Biological Evaluation

#### Cell-free sEH Inhibitory Assay

Human recombinant soluble epoxide hydrolase inhibitor screening assay kit was employed in order to evaluate the enzyme inhibitory potential of the selected compounds using PHOME as a substrate. Mechanistically the epoxide moiety of PHOME gets hydrolyzed by the enzyme with the release of cyanohydrins and further cyanohydrins decompose into cyanide ion and 6-methoxy -2-naphthaldehyde showing high fluorescence which is analyzed at an excitation and emission wavelength of 330 nm & 465 nm. The experiment was performed in triplicates employing enzyme, substrate and the test compounds. The reaction proceeded with the addition of 5 microl of substrate in all wells followed by vigorous shaking of micro-well plate for duration of 10 sec. Incubation period was maintained at 25 °C for 15 minutes in the micro-plate reader (Synergy H1 Biotek) and IC_50_ of AUDA and test compounds was calculated and analyzed.

#### Rat aortic ring based vasodilation assay

Healthy male wistar albino rats (150–250 gm) were placed in polypropylene cages (2/cage) and maintained in 12 h light/dark cycle at temperature of 23 ± 0.5 °C with 55 ± 5.0% relative humidity. The rats had free access to water and feed *ad libitum*. All the procedures employed in the present study were in accordance to CPCSEA (Committee for the Purpose of Control and Supervision of Experiments on Animals) guidelines and regulations. The experimental protocol used in the present study was executed with prior approval of Institutional animal ethical committee (IAEC) of the Banasthali Vidyapith, Rajasthan India (Ref No. BU/BT/3427/16-17).

Rats were sacrificed by high doses of anesthesia and the thoracic aorta was quickly removed and immediately placed in a petri dish containing physiological salt solution (PSS) of the following composition (mM: NaCl 122; KCl 4.7; NaHCO_3_ 15.5; KH_2_PO_4_ 1.2; MgCl_2_ 1.2; CaCl_2_ 2.0; glucose 11.5; EDTA 0.026; pH 7.4). The adhering fat and connective tissue were removed within 15 minute. The aorta was cut into 2–3 mm rings which were used for each set of individual triplicate experiments. The rings were mounted between two stainless steel hooks in a 25-ml jacket organ bath filled with PSS buffer at pH 7.4. One end of hook was connected to the aerator tube and another end connected to force transducer for the measurement of changes in isometric contraction. The PSS buffer in organ bath was continuously bubbled with 95% O_2_–5% CO_2_ gas mixture and its temperature was maintained at 37 °C. Each aortic ring progressively stretched under a passive tension of 2 g before adding any drug and the aortic ring was stabilized for 90 minutes^26,27^.

Post equilibration period, the ring was pre-contracted with 80 mM KCl solution to check its viability. The rings were exposed to cumulative concentration of phenylephrine (10^−4^ to 10^−8^ M) until a stable and sustained contraction was achieved. The effect of each concentration was recorded on organ bath Iworx and was allowed to stabilize before the addition of the next concentration. After this the tissue was incubated with cumulative concentration of reference drug AUDA (10^−4^ to 10^−9^nM) until the maximal relaxation response was obtained. Analogs to procedure used for AUDA the vasodilatory potential of test compounds were recorded and analyzed.

#### *In-vitro* Cytotoxicity assay

In order to examine safety of the selected compounds, MTT (3-(4, 5-Dimethylthiazol-2-yl)-2,5-diphenyltetrazolium bromide) assay was performedusing human Wharton-Jelly derived Mesenchymal Stem Cells (hWJ-MSC). The hWJ-MSC was procured from HiMedia Laboratories and maintained on alpha-MEM (Invitrogen Corporation, USA) supplemented with 12% MSC qualified FBS (Invitrogen Corporation, USA), 1X GlutaMax (Invitrogen Corporation, USA). 1X Antibiotic/Antimycotic (Invitrogen Corporation, USA) was also added to prevent microbial contamination in T25 flasks (Nunc International, USA).

MTT (3-(4,5-Dimethylthiazol-2-yl)-2,5-diphenyltetrazoliumbromide) assay protocol was performed using 96 well plates. A density of 1 × 10^4^ WJ-MSCs were seeded in each wells and incubated in humidified incubator (5% CO_2_ and 37 C) for 24 h. After incubation, cells were exposed to different concentrations (10–40 M) of the test compounds for 24 h, 48 h and 72 h followed by addition of MTT (10-l/well; 5 mg/ml in sterile PBS) and re-incubation of 4 h. Further, the supernatants were removed and replaced with 100 µL DMSO to solubilize the resulting purple formazan crystals produced from metabolically viable cells. Absorbance of the resulting solution was recorded at 550 nm using multi-well microplate reader (Synergy HT, Bio-Tek, USA)^16^. Experiments were performed in triplicates and results were averaged.

## Electronic supplementary material

Supplementary information
