# Supplementary material for: Discovery of Novel Soluble Epoxide Hydrolase Inhibitors as Potent Vasodilators
Source: Sci Rep. 2018 Oct 2;8:14604. doi: 10.1038/s41598-018-32449-4 (PMC6168526; doi:10.1038/s41598-018-32449-4)
Supplement: Supplementary file 1 — Supplementary information [file 41598_2018_32449_MOESM1_ESM.doc]

**Supplementary Material**

**Discovery of Novel Soluble Epoxide Hydrolase Inhibitors as Potent Vasodilators**

Neetika Tripathi^1,+^, Sarvesh Paliwal^1,*^, Swapnil Sharma^1,+^, Kanika Verma^1,+^, Ritika Gururani^1,+^, Amrita Verma^1,+^, Monika Chauhan^1,+^, Akanksha Tiwari^1,+^, Aarti Singh ^1,+^, Dipak Kumar^2,+^, Aditya Pant^2,+^.

^1^Department of Pharmacy, Banasthali University, P. O. Banasthali-304022, Rajasthan, India.

^2^Indian Institute of Toxicology Research, Mahatma Gandhi Marg, Post Box No- 80, Lucknow-226001, U.P, India

^*^[paliwalsarvesh@yahoo.com](mailto:paliwalsarvesh@yahoo.com)

^+^these authors contributed equally to this work

**Table S1** Statistical results of the best 10 pharmacophore hypothesis.

| **Hypo** | **RMSD** | **Correlation** | **Weight** | **Configuration** | **Feature** |
| --- | --- | --- | --- | --- | --- |
| 1 | 0.62 | 0.95 | 0.94 | 12.64 | 2HBA 1HY 1RA |
| 2 | 0.86 | 0.91 | 0.99 | 12.64 | 2HBA 1HY 1RA |
| 3 | 1.00 | 0.89 | 1.27 | 12.64 | 2HBA 1HY 1RA |
| 4 | 1.08 | 0.86 | 0.77 | 12.64 | 2HBA 1HY 1RA |
| 5 | 1.13 | 0.85 | 0.90 | 12.64 | 2HBA 1HY 1RA |
| 6 | 1.12 | 0.85 | 1.12 | 12.64 | 2HBA 1HY 1RA |
| 7 | 1.12 | 0.85 | 1.10 | 12.64 | 2HBA 1HY 1RA |
| 8 | 1.14 | 0.86 | 1.60 | 12.64 | 2HBA 1HY 1RA |
| 9 | 1.22 | 0.82 | 0.78 | 12.64 | 2HBA 1HY 1RA |
| 10 | 1.22 | 0.78 | 0.96 | 12.64 | 2HBA 1HY 1RA |

**Table S2** Results of various parameters calculated during Güner-Henry scoring.

| **Serial No** | **Parameter** | **sEHinhibitors** |
| --- | --- | --- |
| 1 | Total molecules in database (D) | 311 |
| 2 | Total number of active in database (A) | 238 |
| 3 | Total hits (Ht) | 116 |
| 4 | Active hits (Ha) | 105 |
| 5 | . %Yield of actives[(Ha/Ht)×100] | 90.51 |
| 6 | Enrichment factor (E) [(Ha×D)/(Ht×A) | 1.18 |
| 7 | False positives (Ht-Ha) | 11 |
| 8 | Goodness of hit score | 0.67 |

**Table S3** List of selected hits from Maybridgeand NCI database.

| **S. No.** | **Name of hits** | **Estimated Value** | **Fit Value** |
| --- | --- | --- | --- |
| 1 | HTS 04151 | 0.41 | 7.48 |
| 2 | HTS 00684 | 0.72 | 7.25 |
| 3 | NSC 10203 | 0.98 | 7.10 |
| 4 | NSC 13005 | 1.095 | 7.06 |
| 5 | NSC 10020 | 3.93 | 6.50 |
| 6 | NSC 9336 | 5.2 | 6.38 |
| 7 | KM 09759 | 8.63 | 6.16 |
| 8 | BTB 06967 | 12.63 | 5.99 |

**Table S4** Structure and biological activity of the non urea derivatives

| **NON-UREA DERIVATIVES** | | | |
| --- | --- | --- | --- |
| **S.No** | **Name of the compound** | **Structure** | **IC_50_(nM)** |
| **1** | **5a** |  | **0.8** |
| **2** | **5b** |  | **3.9** |
| **3** | **5c** |  | **1.2** |
| **4** | **5d** |  | **8.2** |
| **5** | **6a** |  | **88** |
| **6** | **6b** |  | **2.5** |
| **7** | **6c** |  | **390** |
| **8** | **6d** |  | **28** |
| **9** | **6e** |  | **93** |
| **10** | **6f** |  | **55** |
| **11** | **7** |  | **15000** |
| **12** | **8** |  | **22** |
| **13** | **9** |  | **3600** |
| **14** | **11** |  | **110** |
| **15** | **16b** |  | **23** |
| **16** | **16c** |  | **530** |
| **17** | **17b** |  | **49** |
| **18** | **17c** |  | **48** |
| **19** | **19a** |  | **310** |
| **20** | **19b** |  | **270** |
| **21** | **19c** |  | **185** |
| **22** | **19d** |  | **11** |
| **23** | **20a** |  | **210** |
| **24** | **20b** |  | **1110** |
| **25** | **20c** |  | **1540** |
| **26** | **20d** |  | **685** |

**Table S5** Experimental and predicted activity values of training set compounds

| **Compound name** | **Experimental activities** | **Predicted activities** |
| --- | --- | --- |
| 11 | 110 | 139.593 |
| 16b | 23 | 50.384 |
| 16c | 530 | 273.155 |
| 17b | 49 | 31.148 |
| 17c | 48 | 33.682 |
| 19b | 270 | 461.209 |
| 19c | 185 | 161.123 |
| 19d | 11 | 11.191 |
| 20a | 210 | 493.199 |
| 20d | 685 | 899.182 |
| 5a | 0.8 | 1.07 |
| 5b | 3.9 | 4.533 |
| 5c | 1.2 | 2.28 |
| 5d | 8.2 | 15.751 |
| 6a | 88 | 23.547 |
| 6b | 2.5 | 1.397 |
| 6d | 28 | 29.869 |
| 7 | 15000 | 2743.27 |
| 8 | 22 | 48.906 |
| 9 | 36 | 20.021 |

**Table S6** Experimental and predicted activity values of test set compounds

| **Compound name** | **Experimental activities** | **Predicted activities** |
| --- | --- | --- |
| 19a | 310 | 35.38 |
| 20b | 1110 | 1208.32 |
| 20c | 1540 | 1169.57 |
| 6c | 390 | 51.422 |
| 6e | 93 | 31.158 |
| 6f | 55 | 1.748 |
